# FRAX-based national survey of osteoporotic fracture risk, related knowledge, and associated factors, among Egyptian adults aged 40 years and older, 2025

**DOI:** 10.1038/s41598-026-40443-4

**Published:** 2026-04-29

**Authors:** Doaa I. Omar, Samar A. Amer, Shimaa Gamal Mohammed, Ahmed Abdelwahab Elsheik, Saad Abdelrahim Shoulah, Ibrahim Saeed Aljulaymi, Ibrahim Metwally Dewir, Mai Magdy Anwer

**Affiliations:** 1 Department of Community, Environmental and Occupational Medicine, Faculty of Medicine, Benha University, Benha, Egypt; 2 Department of Public Health and Community Medicine, Faculty of Medicine, Zagazig University, Zagazig, Egypt; 3 Department of Orthopedic Surgery, Faculty of Medicine, Benha University, Benha, Egypt; 4 Department of Physical Therapy, College of Applied Medical Sciences, Taif University, Taif, 21944 Saudi Arabia; 5 Department of Physical Therapy, Cairo University Hospitals, Cairo, Egypt

**Keywords:** Bone Mineral Density (BMD), Dual X-ray Absorptiometry (DXA), Fracture Risk Assessment (FRAX), Osteoporosis Knowledge Assessment Test (OKAT), Osteoporosis Fracture Knowledge Screening Egypt, Diseases, Endocrinology, Health care, Medical research, Risk factors

## Abstract

Osteoporosis is a silent, progressive condition; however, it can be prevented and managed. The assessment of 10-year fracture risk can predict long-term osteoporotic fracture risk. This study was designed to examine osteoporotic fracture risk, its associated factors, and related knowledge among Egyptian adults aged 40+ from the 1^st^ of April 2025 to the 1^st^ of July 2025. This national survey included 2,242 participants over 40 years old from different regions in Egypt, using a questionnaire with five parts, including the OKAT questionnaire and the online FRAX tool, which was filled out by an interviewer. The mean age of participants was 50.6 years, ranging from 40 to 90 years, and the mean BMI was 29.9 (± 5.6) kg/m². 2% and 4.1% were at high risk for major and hip fractures, respectively. The risk of hip and major osteoporotic fractures was significantly associated with long-term cortisone use, smoking, how long someone has smoked, higher consumption of soft or energy drinks, as well as early menopause and using hormonal contraceptives among the women in the study. 58.7% had a poor knowledge level. The OKAT score showed a negative correlation with fracture risks, significant only for hip fracture (*p* = 0.007). A strong positive correlation was found between major and hip fracture risks. Most studied adult Egyptian participants had significant knowledge gaps and misconceptions, a high prevalence of risk factors, and inadequate preventive practices. Therefore, it is important to emphasize the necessity for targeted educational interventions and screening through reliable sources to reduce the future burden of osteoporosis.

**Supplementary Information:**

The online version contains supplementary material available at 10.1038/s41598-026-40443-4.

## Introduction

Osteoporosis (OP) is a progressive disorder, often silent disorder that can be prevented and managed. It is a bone disease characterized by low bone density and damage to the bone structure, which increases the chance of weak bones and fractures from even small falls or injuries^1^. Osteoporosis is a silent condition. However, it can also be prevented and managed. There are two types of risk factors for osteoporosis: non-modifiable and modifiable. Modifiable risk factors include Body Mass Index, smoking, alcohol consumption, falls, prolonged use of corticosteroids, and proton pump inhibitors^2^.

Osteoporosis is predicted to develop in Egyptians over 50 years old. Although osteopenia is projected to affect 53.9% of Egyptian women and 26% of Egyptian men, osteoporosis prevalence in Egypt is estimated to be 21.9% for males and 28.4% for women. Half of all postmenopausal women with osteoporosis are predicted to suffer an osteoporosis-related fracture in their lifetime; of these, 25% will suffer a vertebral deformity, and 15% will sustain a hip fracture^3^.

Osteoporosis is identified by measuring Bone Mineral Density (BMD) using Dual X-ray Absorptiometry (DXA). About two-thirds of bone strength is determined by areal BMD. As BMD decreases, the fracture risk rises^4^.

For osteoporotic fractures, there are at least two main causes: low bone density and the frequency and type of falls. Interventions aimed at changing these risk factors can help prevent fractures. Additionally, risk factors can be used to identify individuals at the highest risk who would benefit from preventive therapy before fractures happen. Low bone density is a significant risk factor for osteoporotic fractures, and its link to fracture risk is discussed elsewhere in this issue^5^.

There is convincing evidence that assessment of the 10-year probability of fracture risk can predict the long-term osteoporotic fracture risk in both women and men, whereas BMD assessment can predict short-term osteoporotic fracture risk. Furthermore, FRAX screening is less expensive than DXA scanning (both in terms of the time and effort patients and the healthcare system must invest)^6^.

The risk of fragility fracture rises significantly at the age of 60. Since it would take a long time to reduce this risk through proper management of osteoporosis or behavioral risk factors, it is recommended that early detection of fragility fractures in Egyptian postmenopausal women and men begin at the age of 50^6^.

Understanding knowledge and attitudes about osteoporosis among at-risk males and females, early osteoporosis prediction, and preventative health activities are crucial. Subsequently, this study aimed to identify osteoporosis-related knowledge and assess the 10-year risk of osteoporotic fracture among Egyptian adults, both males and females aged 40 years and above, based on the FRAX assessment tool and its associated risk factors during the period from the 1^st^ of April  2025 to the 1^st^  of July 2025.

## Methodology

### Study design and duration

This was a national cross-sectional survey. The survey was conducted from April 1, 2025, to July 1, 2025. The study followed all relevant guidelines and regulations.

### Participants

The inclusion criteria were being an Egyptian adult aged 40 years and over who agreed and agreeing to participate in the study. Individuals under 40, non-Egyptian, or unwilling to provide informed permission were excluded from the study. Individuals with cognitive impairment, severe psychiatric disorders, or communication difficulties that could affect questionnaire responses were excluded. The final study excluded subjects with incomplete FRAX calculations or insufficient osteoporosis knowledge data. Individuals with a history of malignancy, including bone metastases or other conditions leading to pathological fractures not associated with osteoporosis, were excluded to reduce the potential for misclassification of fracture risk.

Individuals aged 40 and above should undergo early fracture risk assessment, as they are recognized during the preclinical phase of fracture risk accumulation, considering that bone loss and fracture risk factors initiate prior to the manifestation. After reaching peak bone mass in the late 20 s, bone mineral density gradually declines, becoming more noticeable in the fourth decade, especially with the presence of risk factors. Although fragility fractures increase dramatically beyond 60, the pathophysiological processes often start 10–20 years before. Therefore, early fracture risk assessment is necessary for individuals aged 40 and older, as it is standard practice to modify behaviors before peak fracture incidence; this process identifies high-risk participants. Further specialized consultations and investigations facilitate timely referrals and preventative measures before irreversible bone loss and fractured episodes occur^7,8^.

### Sampling techniques and sample size

Data collection utilized a combination of convenient and snowball sampling techniques. However, to ensure national representation, researchers stratified the study population geographically during the selection of collaborating students and data collectors. We used proportional allocation based on regional population size to reduce sample bias and capture socio-demographic and health system variation. The Greater Cairo Region, Delta Region, Alexandria Region, Suez Canal Region, North Upper Egypt Region, Asyut Region, and South Upper Egypt Region received samples. Moreover, the study involved about one-quarter of the different Egyptian governorates^9^.

**The sample size** was calculated according to the following formula: *n* = Z² p (1-p)/d², *where n is* the minimum sample size. Z is the standard normal variate that equals 1.96, and P is the prevalence rate of osteoporosis in Egypt (28.4% in women and 21.9% in men) according to^6^. With a 95% level of confidence and a d^2^ (level of precision) of 0.05. The calculated sample size for women was 316, and 263 for men, considering 30% non-respondents, so the total sample was 753. The study recruited a total of 2242 participants, achieving a response rate of 80% through continuous efforts.

### Data collection method

The interviewer-administered questionnaire (Google Form) was used by the collaborating undergraduate medical students from Benha Faculty of Medicine. After conducting training sessions, students were trained regarding osteoporosis, FRAX, and the study questionnaire. FRAX, and the study questionnaire’s different compartments. The students were trained on how to use the Egyptian FRAX website and how to access it. The students collected the data by face-to-face interviews with eligible participants.

### Data collection tool

#### Preparation and validation of the data collection tool were conducted to ensure its effectiveness

The questionnaire was prepared based on previous studies^2,6,10–12^. A pilot study was conducted with 35 adults over the age of 40 to assess the questionnaire’s comprehension and clarity, as well as to test its validity by calculating Cronbach’s alpha, which was found to be 0.81; those participants were excluded from the final analysis.

#### The structure of the data collection tool

The study questionnaire was composed of five sections, as follows:

1. **Sociodemographic characteristics**: age, sex, governorate, residence, education, marital status, and occupation.
2. **Risk characteristics **of participants include having chronic diseases, personal and family history of osteoporotic fractures, women’s obstetric history (like age of menopause and number of children), lifestyle habits (such as smoking, diet including dairy intake, sun exposure, physical activity, and consumption of coffee, tea, and soft drinks), medications taken by participants, and personal body changes like having kyphosis or being shorter.
3. **Osteoporosis Knowledge Assessment Tool (OKAT) **– Arabic Version: The Arabic version of the Osteoporosis Knowledge Assessment Tool (OKAT), previously validated in Syria (2013) and utilized in an Egyptian study, was employed in this study. The instrument was obtained through direct written correspondence with the author of the Egyptian study, with formal permission granted via email (request sent March 11, 2025; permission received March 28, 2025; confirmation March 29, 2025). The questionnaire link and authorization for academic use were provided by the corresponding author, and documentation of this correspondence has been retained and submitted as supplementary material^6,11^.
  The Arabic OKAT was used in its original format, with no structural modifications. Only minor formatting adjustments were made to standardize the layout for electronic data collection, without altering item content or scoring.
  The OKAT consists of 20 items: the first 12 assess knowledge about osteoporosis, the next 4 assess attitudes, and the final 4 evaluate preventive factors. Each item offers three response options (“yes,” “no,” “I do not know”). Responses were scored with one point for each correct answer and zero for incorrect or “do not know” answers. The total score ranges from 0 to 20 and was converted to a percentage (score divided by 20). Participants’ knowledge levels were categorized using a 60% cutoff.
4. **The anthropometric measurements**: weight, height, and BMI. **5)** **The Egyptian Fracture Risk Assessment tool (Egyptian FRAX tool) **evaluates the 10-year risk of osteoporotic fractures based on factors such as age, sex, weight, height, previous fractures, parents’ hip fractures, current smoking status, glucocorticoid use (for 3 months), rheumatoid arthritis, secondary osteoporosis, and alcohol intake^11^. Online access to the Egyptian FRAX site and previously mentioned data was filled, resulting in Hip fracture and major osteoporotic fracture scores.

The cutoffs for high fracture risk were obtained from Egyptian reports (the position statement of the Egyptian Academy of Bone Health, Egyptian Rheumatology, and Rehabilitation). Egyptians were classified as high risk of osteoporotic fracture when the 10-year FRAX probability of major osteoporosis risk was ≥ 20% or hip fracture risk was ≥ 3%^6^.

### Statistical analysis

There was no missing data, as all the questionnaire items were filled out on a Google form, and all questions were mandatory before submission. The Statistical Package for Social Science version 27 was used to code and analyze the data collected.

Categorical variables were presented with frequencies and percentages. For quantitative data, the normality of distribution was assessed using the Kolmogorov-Smirnov test. Since the data were not normally distributed, variables were summarized using Median (range) to describe central tendency and dispersion. Mean ± standard deviation (SD) was provided as an additional measure of average and variability.

The Mann-Whitney test was used for comparisons between two independent groups, and the Kruskal-Wallis’s test was used for comparisons among more than two groups. Post hoc comparisons were performed using Duncan’s multiple range test to identify pairwise differences between groups. A *p* value < 0.05 was considered statistically significant. Spearman’s correlation was conducted between quantitative variables. All statistical tests were two-tailed, and a p-value of less than 0.05 was considered statistically significant.

Importantly, the prevalence of high-risk FRAX categories in the study population was very low. Under such conditions, multivariable regression modeling would be prone to sparse-data bias, unstable estimates, and limited interpretability. Consequently, bivariate analyses were considered methodologically appropriate for this exploratory phase.

### Ethical approval

Informed verbal consent was obtained from all participants after clarifying the objectives of the study, confidentiality of data, and voluntary involvement. Ethical approval from the Research ethics committee at Benha Faculty of Medicine, with the following reference number (RC 1–3−2025).

During data collection, individuals with increased FRAX scores were notified of their estimated fracture risk and recommended, as general advice, to seek further clinical examination in accordance with national and international osteoporosis recommendations. These recommendations constituted ethical research practices and were not regarded as study interventions.

## Results

### Demographic and general characteristics of Egyptian participants

A total of 2242 Egyptian participants were included. The mean age was 50.6 years, ranging from 40 to 90 years, and the mean (SD) of BMI was 29.9 (± 5.6) kg/m². The majority were married (85.1%) and university graduates (66.5%), with 38.2% working in governmental jobs. Most participants reported having sufficient income (77.8%) and were from rural areas (58.4%), nearly equally distributed between males (51.7%) and females (48.3%) (Table 1).

**Table 1:** **Demographic characteristics and their associations with knowledge, major, and hip fracture risk among the Egyptian participants.**

The variables	Total	Knowledge	Major fracture	Hip fracture
	N = 2242	Median (Mean ± SD) (range)	Median (Mean ± SD) (range)	Median (Mean ± SD) (range)
	F (%)			
**Sex**				
Male	1158(51.7)	10 (9.9 ± 4.1) (0–18)	2.6 (3.5 ± 2.8) (0.1–28.7)	0.2 (0.6 ± 1.5) (0.0–22)
Female	1084(48.3)	11(10.7 ± 3.7) (0–18)	2.6 (4.2 ± 4.5) (0.8–40)	0.2 (0.74 ± 1.9) (0.0–29)
*P*-value^•^		0.003^*^	0.59	0.001^*^
**Residence **				
Rural	1309(58.4)	11(10.2 ± 3.9) (0–18)	2.6(3.9 ± 3.8) (0.1–40.8)	0.2(0.64 ± 1.5) (0–29)
Urban	933(41.6)	11(10.43 ± 3.9) (0–18)	2.6(3.8 ± 3.5) (0.1–30)	0.2(0.7 ± 1.9) (0–22)
*P*-value^•^		0.12	0.81	0.43
**Marital status **				
Single	107 (4.8)	10(9.79 ± 4.1) (0–17)	2.4(2.68 ± 1.7) (0.1–9.6)^a^	0.1(0.27 ± 0.35) (0–2.2)
Married	1909 (85.1)	11(10.4 ± 3.9) (0–18)	2.5(3.6 ± 3.3) (0.1–40.8)^a^	0.2(0.6 ± 1.4) (0–22)^a^
Widow/divorced	226 (10.1)	10(9.73 ± 4.3) (0–18)	5.3(6.8 ± 5.57) (0.7–35)^b^	0.8(1.8 ± 2.9) (0–29)^b^
*P*-value^•^^•^		0.76	< 0.001^*^	< 0.001^*^
**Age (y)**		r(*P*)	r(*P*)	r(*P*)
Mean ± SD	55.9 ± 10.9	−0.081 (< 0.001^*^)	0.42(< 0.001*)	0.403(< 0.001*)
Range	40–90			
40-<50 y	856(38.2)	11.00; 10.51 ± 3.79 (0.00–18.00)	2.20; 4.22 ± 7.86	0.10; 0.47 ± 6.57 (0.00–3.6)
50-<60y	749 (33.4)	11.00; 10.56 ± 3.94 (0.00–18.00)	3.20; 5.69 ± 7.5	0.30; 1.11 ± 10.09 (0.00–13)
60-<70y	509 (22.7)	10.00; 9.47 ± 4.21 (0.00–17.00)	5.30; 8.86 ± 13.06	0.85; 2.84 ± 14.36 (0.00–22)
>70 or equal	128(5.7)	9.00; 8.69 ± 4.52 (0.00–17.00)	6.20; 9.57 ± 12.70	2.10; 5.72 ± 22.39 (0.00–29)
*P*-value^•^^•^		< 0.001*	< 0.001*	0.068
**Egyptian planning region**				
Greater Cairo Region	595 (26.0)	11 (10.4 ± 3.9) (0–18)	2.6 (4.3 ± 6.9) (0.9–100)	0.2(1.4 ± 13.2) (0–22)
Delta Region	824 (36.0)	11(10.9 ± 3.2) (2–16)	2.5(4.2 ± 5.3) (1.1–28)	0.2 (0.61 ± 1.1) (0–5.1)
Alexandria Region	252 (11.0)	11 (10.2 ± 4.3) (0–18)	2.6 (3.7 ± 3.7) (0.1–40)	0.2(0.61 ± 1.3) (0.0–13.0)
Suez Canal Region	183 (8.0)	11(10.23 ± 3.6) (0–18)	3(4.2 ± 4.1) (0–35)	0.2(0.8 ± 2.4) (0–29)
North Upper Egypt Region	160 (7.0)	10(9.7 ± 4.4) (0–17)	2.6 (3.5 ± 3.1) (0.1–27)	0.2(0.5 ± 0.84) (0–5.1)
Asyut Region	114 (5.0)	11 (10.7 ± 4.4) (0–16)	3 (4.6 ± 3.8) (1.4–18)	0.35(0.65 ± 0.8) (0.1–3.6)
South Upper Egypt Region	161 (7.0)	9 (9.5 ± 4.1) (0–16)	2.9(4.6 ± 4.4) (0.8–27)	0.4(0.75 ± 1.14) (0–5.2)
*P-*value^•^^•^		0.149	0.165	0.663
**Level of education**				
Illiterate	102 (4.5)	8(7.9 ± 4.5) (0–17)^a^	5.2(6.7 ± 5.1) (1.6–35)^a^	0.8(1.9 ± 3.5) (0–29)^a^
Primary	95 (4.2)	9(8.8 ± 4.4) (0–17)^a^	5(6.23 ± 5.2) (0.1–29)^b^	0.7(1.65 ± 2.3) (0–15)^a^
Preparatory	59 (2.6)	9(8.47 ± 4.38) (0–16)^a^	3.8 (4.9 ± 4.1) (0.9–27)^c^	0.6(0.9 ± 1.1) (0–5)^a^
Secondary	496 (22.1)	10(10.05 ± 4.0) (0–17)^b^	2.7(3.9 ± 3.8) (0.1–40.8)^d^	0.2 (0.58 ± 1.16) (0–12)^b^
University	1490 (66.5)	11(10.6 ± 3.8) (0–18)^c^	2.7 (3.4 ± 3.3) (0.1–32)^e^	0.2 (0.54 ± 1.6) (0–22)^c^
*P*-value^•^^•^		< 0.001^*^	< 0.001^*^	< 0.001^*^
**Current job**				
Manual	84 (3.7)	10(9.3 ± 4.2) (0–17)	2.9(3.9 ± 2.5) (0.1–12)	0.2(0.6 ± 0.9) (0–4.5)
Housewife/not working	540 (24.1)	10(10.04 ± 3.9) (0–18)	2.6(4.5 ± 4.7) (0.8–35)	0.2(0.9 ± 2.5) (0–29)
Governmental	856(38.2)	11(10.8 ± 3.8) (0–18)	2.5(3.5 ± 3.35) (0–40.8.8)	0.2(0.4 ± 0.87) (0.0–13)
Private	509 (22.7)	11(10.1 ± 4.2) (0–18)	2.3(3.0 ± 2.8) (0.7–28.7)	0.1(0.38 ± 1.2) (0–22)
Retired	253 (11.3)	10 (9.9 ± 3.9) (0–17)	4.1(5.7 ± 3.9) (1.8–28)	0.9(1.6 ± 2.3) (0–22)
*P-*value^•^^•^		< 0.001^*^	< 0.001^*^	< 0.001^*^
**Income **				
Insufficient	146 (6.5)	11(10.1 ± 4.1) (0–17)	2.7(3.4 ± 2.1) (0.55–12.55)	0.2(0.57 ± 1.3) (0–7.5)
Sufficient	1745 (77.8)	11(10.3 ± 3.9) (0–18)	2.6 (3.9 ± 3.7) (0.10–40.8)	0.2(0.6 ± 1.5) (0–29)
Sufficient and more	351 (15.7)	11(10.5 ± 4.2) (0–18)	2.7(4.1 ± 4.2) (0.1–30)	0.2 (0.8 ± 2.2) (0–22)
*P*-value^•^^•^		0.51	0.62	0.39
**BMI **		r(*P*)	r(*P*)	r(*P*)
Mean ± SD	29.9 ± 5.6	0.12(< 0.001^*)^	−0.037(0.107)	0.043(0.055)
Range	14.1–69.5			
**Number of children**		r(*P*)	r(*P*)	r(*P*)
Mean, SD	3.47 ± 1.1	−0.081(0.41)	0.108(< 0.001^**^)	0.110(< 0.001^*)^
(Range)	(0–12)			
**Comorbidity**				
None/others	1698(75.7)	11(10.4 ± 3.9) (0–18)	2.5 (3.4 ± 2.9) (0.1–35)	0.2 (0.5 ± 1.4) (0–29)
RA	210(9.4)	10 (9.9 ± 4.1) (0–17)	4(5.9 ± 5.8) (0.9–40.8)	0.4(1.4 ± 2.7) (0–22)
Chronic gastritis	59(2.6)	11 (10.8 ± 3.4) (2–16)	2.7 (4.6 ± 4.5) (0.9–28.7)	0.2(0.8 ± 1.4) (0–7.4)
Cancer	4(0.2)	1.5 (9.0 ± 3.4) (4–11)	4.3 (8.3 ± 9.1) (2.6 ± 22)	0.6(3.6 ± 6.3) (0.2–13)
Malnutrition	26(1.2)	9(9.9 ± 3.5) (4–16)	2.4(3.9 ± 5.7) (1.1–28)	0.1 (0.4 ± 1.04) (0–5.1)
Liver failure	8(0.8)	9(8.6 ± 2.6) (5–12)	6.4 (5.5 ± 3.8) (1–10)	0.4 (1.1 ± 1.29) (0–3.4)
Renal failure	16(0.7)	10(10.8 ± 2.3) (8–16)	6.7(7.4 ± 6.6) (1.10–27)	0.9(1.8 ± 1.9) (0–5.2)
Thyroid disorders	34(1.5)	10.5(9.7 ± 4.04(0–17)	3.7 (4.7 ± 2.9) (1.2–12)	0.4(0.7 ± 0.92) (0–4.5)
Parathyroid disorder	9(0.4)	9(8.6 ± 2.6) (5–12)	6.4(5.5 ± 3.8) (1–10)	0.4 (1.1 ± 1.3) (0–3.4)
*P*value^•^^•^		0.429	< 0.001^*^	< 0.001^*^

^•^Mann-WhitneyU ^••^Test The Kruskal-Wallis Test.

*Significant at ≤ 0.01 Values sharing the same superscript letter are not significantly different, whereas different letters denote statistically significant differences (*p* < 0.05).

#### The associations between demographic determinants and the OKAT score

Females demonstrated significantly higher knowledge levels compared to males (*p* = 0.003). Educational level was strongly associated with knowledge, with university graduates scoring higher than those with lower education (*p *< 0.001). Age showed a negative association; older participants had lower knowledge scores (*p *< 0.001). Place of residence (rural/urban) and income did not show a significant association or affect the knowledge levels (Table 1).

#### The knowledge-related context: sources, items, and the OKAT knowledge scores classification among the Egyptian participants

The participants showed good awareness of some key facts, such as osteoporosis increasing fracture risk (87.1%), the benefits of physical activity (77.7%), the protective role of peak bone mass (79.4%), and family history (61.0%).

However, the majority didn’t know about essential osteoporosis facts, e.g., white women are at highest risk of fractures (61.1%); hormone therapy prevents bone loss after menopause (59.4%); there are no effective treatments available in Egypt (56.7%); postmenopausal bone loss within 10 years was 53.8%; and 52.1% did not recognize high salt intake as a risk factor.

Moreover, several misconceptions were also evident, e.g., 74.4% incorrectly believed that osteoporosis causes symptoms before fractures, 46.3% thought it is more common in men, and 31.8% assumed calcium supplements alone could prevent bone loss. These false beliefs may delay early detection and proper prevention (Table 2).

**Table 2:** **The knowledge related context: Sources, items, and classification among the Egyptian participants.**

**Variable **	**F (%)**		
**The main source of knowledge **			
TV or radio	432(19.3)		
Family or friend	472(21.0)		
Physician	272(12.1)		
Scientific	283(12.6)		
Social media	783(36.9)		
**Aware of the osteoporosis disease **	2077 (92.6)		
**The knowledge related questions**	**Yes **	**No **	**Don’t know **
1. Osteoporosis leads to an increased risk of bone fractures	**1953(87.1)**	66(2.9)	223(9.9)
2. Oosteoporosis usually causes symptoms (e.g., pain) before fractures occur .	1668 (74.4**)**	**120(5.4)**	454(20.2)
3. Having a higher peak bone mass at the end of childhood gives no protection against the development of osteoporosis in later life.	**1781(79.4)**	118(5.3)	343(15.3)
4. Osteoporosis is more common in men .)	290(12.9**)**	**1037(46.3)**	915(40.8)
**5.**Cigarette smoking can contribute to osteoporosis .	**1320(58.9)**	155(6.9)	767(34.2)
6. White women are at highest risk of fracture as compared to other races	**440(19.6)**	433(19.3)	1369(61.1)
**7.**A fall is just as important as low bone strength in causing fractures	**1127(50.3)**	359(16.0)	756(33.7)
8. By age 80, most women have osteoporosis	**1457 (65.0)**	80(3.6)	705 (31.4)
**9.**From age 50, most women can expect at least one fracture before they die	**1252(55.8)**	168(7.5)	822(36.7)
**10.**Physical activity is beneficial for osteoporosis	**1742(77.7)**	98 (4.4)	402(17.9)
**11.**It is easy to tell whether I am at risk of osteoporosis by my clinical risk factors	**166(74.3)**	83(3.7)	493(22.0)
**12.**Family history of osteoporosis strongly predisposes a person to osteoporosis	**1368(61.0)**	295(13.2)	579(25.8)
**13.**An adequate calcium intake can be achieved from two glasses of milk a day	**1396(62.3)**	313(14.0)	533(23.8)
**14.**Sardines and broccoli are good sources of calcium for people who cannot take dairy products	**1573 (70.2)**	233(10.4)	436(19.4)
**15.**Calcium supplements alone can prevent bone loss	259(11.6)	**1271(56.7)**	712(31.8)
16. Alcohol in moderation has little effect on osteoporosis	**1543(68.8)**	92(4.1)	607(27.1)
**17.**A high salt intake is a risk factor for osteoporosis.	**826(36.8)**	**247(11.0)**	1169(52.1)
**18.**There is a small amount of bone loss in the 10 years following the onset of menopause	951(42.4)	**84 (3.7)**	1207(53.8)
**19.**Hormone therapy prevents further bone loss at any age after menopause	**746(33.3)**	164(7.3)	1332(59.4)
**20.**There are no effective treatments for osteoporosis available in “Egypt”	**460(20.5)**	510(22.7)	1272(56.7)
**Total knowledge score **	**Means, SD (range) 11.15, 4.2 (0–19)**		
Poor < 60% (< 12)	1315(58.7%)		
Good > 60% (12 or more)	927 (41.3%)		

Bold indicates – the right answer.

**Overall, OKAT knowledge scores** the mean osteoporosis knowledge score was 11.15 out of 20, with a wide range (0–19). More than half of participants (58.7%) were classified as having poor knowledge (< 60% correct answers), while only 41.3% demonstrated good knowledge (≥ 60%) (Table 2).

#### The Association between demographic determinants and the osteoporotic fracture risk score

Widowed and divorced participants were more prone to major and hip fractures compared to married individuals (*p* < 0.001). Advancing age was significantly associated with increased fracture risk (*p* < 0.001). Lower educational attainment and occupational categories such as manual jobs and retirement were linked to higher fracture rates (*p* < 0.001). Comorbidities, including rheumatoid arthritis, renal failure, and thyroid disorders, were significantly associated with greater fracture occurrence (*p* < 0.001). In contrast, income level did not show a significant impact on fracture risk (Table 1).

### Osteoporosis risk factors among the Egyptian studied groups

Most of the 2242 Egyptian participants were non-smokers (80%), did not consume alcohol (99.2%), and had no history of prolonged cortisone use (93.7%). Nearly half (48.3%) reported never consuming soft or energy drinks, while 16.5% of women experienced menopause before the age of 45. About 35.5% of participants reported previous bone fractures, and 10% had a family history of hip fracture due to osteoporosis (Table 3).

**Table 3:** **Distribution of risk factors and their associations with major and hip osteoporotic fracture risk among the studied Egyptian participants.**

**Variable**	**Total **	**Major fracture**	**Hip fracture**
	**N = 2242**	**Median (Mean ± SD; Range)**	**Median (Mean ± SD; Range)**
	**F (%)**		
**Alcohol intake**	Yes: 17 (0.8%)	2.6 (3.8 ± 3.7; 0.1–40.8)	0.7 (1.2 ± 1.8; 0.1–5.1)
	No: 2225 (99.2%)	3.4 (6.4 ± 7.8; 0.8–28)	0.2 (0.7 ± 1.7; 0–29)
***p*-value**		0.94	0.67
**Cortisone intake > 3 months**	Yes: 142 (6.3%)	5.4 (6.8 ± 5.9; 0.6–29)	0.6 (1.4 ± 2.3; 0–15)
	No: 2100 (93.7%)	2.6 (3.7 ± 3.4; 0.1–40.8)	0.2 (0.6 ± 1.6; 0–29)
***p*-value**^•^		< 0.001^*^	< 0.001^*^
**Smoking status**	Non-smoker: 1793 (80.0%)	2.6 (3.8 ± 3.8; 0.1–40.8)^a^	0.2 (0.6 ± 1.7; 0–29)^a^
	Ex-smoker: 136 (6.1%)	3.0 (4.3 ± 3.8; 1–28.7)^b^	0.4 (1.1 ± 2.6; 0–22)^b^
	Current smoker: 313 (14.0%)	2.8 (3.8 ± 3.0; 0.5–20)^b^	0.3 (0.7 ± 1.3; 0–13)^a^
***p*-value**^•^^•^		< 0.001^*^	< 0.001^*^
**Smoking duration (n = 449)**	< 1 y: 22 (4.9%)	4.1 (4.9 ± 3.4; 0.8–12)	0.5 (1.0 ± 1.2; 0.1–4.5)
	1–<5 y: 39 (8.7%)	2.1 (3.2 ± 2.5; 1–12)	0.1 (0.7 ± 1.4; 0–7.5)
	5–10 y: 76 (16.7%)	2.5 (3.6 ± 3.7; 0.8–12)	0.2 (0.5 ± 0.7; 0–5.1)
	>10 y: 301 (69.7%)	3.2 (4.2 ± 3.6; 0.6–28.7)	0.4 (0.9 ± 2.1; 0–22)
***p*-value**^•^^•^		0.001^*^	< 0.001^*^
**Soft/energy drinks consumption**	Never: 1082 (48.3%)	2.8 (4.0 ± 3.7; 0.7–40.8)^a^	0.2 (0.7 ± 1.7; 0–29)^a^
	1x/w: 571 (25.5%)	2.4 (3.7 ± 3.8; 0.9–27)^b^	0.2 (0.6 ± 1.7; 0–22)^b^
	2x/w: 283 (12.6%)	2.6 (3.9 ± 4.1; 0.8–32)^a^	0.2 (0.6 ± 1.5; 0–13)
	3x/w: 157 (7.0%)	2.5 (3.7 ± 3.7; 0.5–28)^a^	0.2 (0.7 ± 2.1; 0–22)
	4x/w: 76 (3.4%)	2.7 (3.9 ± 3.1; 0.1–17)^a^	0.3 (0.5 ± 0.8; 0–4.5)^b^
	≥5x/w: 73 (3.3%)	2.6 (3.4 ± 2.2; 0.1–9.5)^b^	0.2 (0.4 ± 0.4; 0–1.8)^c^
**p-value**^•^^•^		0.01^*^	0.004^*^
**Menopause < 45 y (n = 1050)**	Yes: 173 (16.5%)	4.8 (6.4 ± 5.1; 1.2–27)	0.6 (1.3 ± 1.7; 0–8.3)
	No/not applied: 877 (83.5%)	2.5 (3.7 ± 3.5; 0.1–40.8)	0.2 (0.6 ± 1.7; 0–29)
***p*-value**^•^		< 0.001^*^	< 0.001^*^
**Contraceptives (n = 1050)**	Yes: 408 (38.9%)	2.4 (4.0 ± 4.5; 0.6–40.8)	0.1 (0.6 ± 1.5; 0–22)
	No/not applied: 642 (61.1%)	2.7 (3.9 ± 3.4; 0.1–35)	0.2 (0.7 ± 1.7; 0–29)
***p*-value**^•^		0.002^*^	< 0.001^*^
**Hormonal replacement therapy(n = 1050)**	Yes: 99 (9.4%)	2.5 (5.0 ± 5.8; 0.8–32)	0.2 (0.9 ± 2.6; 0–22)
	No/not applied: 951 (90.6%)	2.6 (3.8 ± 3.6; 0.1–40.8)	0.2 (0.7 ± 1.6; 0–29)
***p*-value**^•^		0.57	0.91
**Secondary osteoporosis**	Yes: 161 (7.2%)	5.1 (6.9 ± 5.9; 1.1–35)	0.6 (2.1 ± 4.4; 0–29)
	No: 2081 (92.8%)	2.5 (3.7 ± 3.4; 0.1–40.8)	0.2 (0.7 ± 1.2; 0–22)
**p-value**^•^		< 0.001^*^	< 0.001^*^
**Previous bone fractures**	Yes: 795 (35.5%)	4.2 (5.6 ± 4.8; 0.7–40.8)	0.5 (1.1 ± 2.4; 0–29)
	No: 1447 (64.5%)	2.3 (2.9 ± 2.6; 0.1–32)	0.1 (0.4 ± 1.1; 0–22)
***p*-value**^•^		< 0.001^*^	< 0.001^*^
**Family history of hip fractures**	Yes: 225 (10.0%)	3.9 (6.3 ± 6.1; 0.6–40.8)	0.3 (1.5 ± 3.9; 0–29)
	No: 1536 (68.5%)	2.5 (3.5 ± 3.0; 0.1–30)	0.2 (0.5 ± 1.1; 0–13)
	Don’t know: 481 (21.5%)	2.8 (4.2 ± 3.8; 0.7–32)	0.3 (0.8 ± 1.4; 0–15)
***p*-value**^•^^•^		< 0.001^*^	< 0.001^*^
**Height/shape changes**	Decrease: 133 (5.9%)	3.9 (5.5 ± 14.1; 0.8–27)^a^	0.5 (1.1 ± 1.4; 0–7.9)^a^
	No change: 1855 (82.7%)	2.5 (3.5 ± 3.1; 0.1–40.8)^b^	0.2 (0.5 ± 1.2; 0–22)^b^
	Kyphosis: 254 (11.3%)	3.9 (6.1 ± 5.9; 0.6–35)^a^	0.5 (1.8 ± 3.7; 0–29)^a^
***p*-value**^•^^•^		< 0.001^*^	< 0.001^*^

^•^Mann-Whitney U Test ^•^^•^The Kruskal-Wallis Test *Significant at ≤ 0.01. Values sharing the same superscript letter are not significantly different, whereas different letters denote statistically significant differences (*p* < 0.05).

#### The association of osteoporosis risk factors with major osteoporotic fracture risk score

Major osteoporotic fracture risk was significantly higher among Egyptian participants with prolonged cortisone intake (*p* < 0.001), smokers compared to non-smokers (p < 0.001), and those with longer smoking duration (*p* = 0.001). Moreover, higher major fracture risk was associated with increased soft/energy drink consumption (*p* = 0.01), secondary osteoporosis (*p *< 0.001), history of bone fractures (*p* < 0.001), family history of hip fracture (*p* < 0.001), and height loss or back curvature (*p < *0.001) (Table 3).

However, higher major fracture scores were found among women with menopause before age 45 (*p < 0.001) and* contraceptive use (*p *= 0.002). Alcohol intake and hormonal replacement therapy showed no significant association with major FRAX scores (Table 2).

#### The Association of osteoporosis risk factors with hip osteoporotic fracture risk score

Hip fracture risk followed a similar pattern as major osteoporotic fracture risk, being significantly elevated in participants with cortisone use (*p* < 0.001), smokers (*p* = 0.001), and longer smoking duration (*p *< 0.001). Frequent consumption of soft/energy drinks was also linked to higher hip fracture scores (*p* = 0.004) (Table 3).

Women with early menopause (*p* < 0.001), contraceptive use (*p *< 0.001), secondary osteoporosis (*p* < 0.001), previous fractures (*p *< 0.001), a family history of hip fracture (*p *< 0.001), and noticeable height loss or spinal curvature (*p* < 0.001) all demonstrated significantly higher hip fracture risks. In contrast, alcohol intake and hormonal replacement therapy did not significantly affect hip fracture outcomes (Table 3).

### Protective behaviors among the studied Egyptian participants

Most Egyptian participants were overweight or obese. (70.1%) didn’t use osteoporosis-specific medications (92.4%). Physical activity was low, as 41.9% reported never exercising, while only 16.1% exercised five times or more per week. Daily milk consumption was uncommon, with nearly 70% of individuals consuming just one serving per day and 10% reporting no milk consumption. Around one-third reported daily sun exposure of 15 min or more, and 6.8% had undergone a DEXA scan (Table 4).

**Table 4:** **Distribution of protective and risk- driven behaviors and their associations with major and hip osteoporotic fracture risk among studied Egyptian participants.**

**Variables**	**Total**	**Major fracture **	**Hip fracture**
	**N = 2242**	**Median (Mean ± SD; Range)**	** Median (Mean ± SD; Range)**
	**F (%)**		
**Osteoporosis medications (other than Ca & Vit D)**	Yes: 169 (7.5%)	3.8 (5.9 ± 5.6; 0.6–35)	0.4 (1.6 ± 3.8; 0–29)
	No: 2072 (92.4%)	2.6 (3.7 ± 3.5; 0.1–40.8)	0.2 (0.6 ± 1.4; 0–22)
***p*-value**^•^		< 0.001^*^	< 0.001^*^
**Vitamin D supplementation**	No: 1583 (70.6%)	2.5 (3.6 ± 3.0; 0.1–32)	0.2 (0.6 ± 1.4; 0–22)
	Irregular: 401 (17.9%)	2.7 (4.2 ± 4.4; 0.1–35)	0.2 (0.8 ± 2.2; 0–29)
	Regular: 257 (11.5%)	3.7 (5.5 ± 5.5; 0.6–40.1)	0.3 (1.1 ± 2.2; 0–22)
***p*-value**^•^^•^		< 0.001^*^	< 0.001^*^
**Calcium supplementation**	No: 1675 (74.7%)	2.5 (3.5 ± 3.1; 0.1–40.8)	0.2 (0.6 ± 1.4; 0–22)
	Irregular: 367 (16.4%)	2.8 (4.5 ± 4.9; 0.6–35)	0.2 (0.9 ± 2.6; 0–29)
	Regular: 200 (8.9%)	4.2 (5.7 ± 4.9; 0.8–27)	0.4 (1.2 ± 1.9; 0–13)
***p*-value**^•^^•^		< 0.001^*^	< 0.001^*^
**Physical activity**	Never: 939 (41.9%)	2.8 (4.2 ± 3.8; 0.1–29)	0.2 (0.8 ± 1.9; 0–22)
	1–2x/week: 570 (25.4%)	2.5 (3.7 ± 3.7; 0.8–35)	0.2 (0.6 ± 1.9; 0–29)
	3–4x/week: 372 (16.6%)	2.6 (3.6 ± 3.4; 0.9–40.8)	0.2 (0.5 ± 1.1; 0–13)
	≥5x/week: 361 (16.1%)	2.6 (3.7 ± 3.9; 0.1–32)	0.2 (0.5 ± 1.0; 0–10)
***p*-value**^•^^•^		0.004^**^	< 0.001^*^
**Milk/derivatives per day**	> 3 servings: 48 (2.1%)	2.6 (3.1 ± 2.0; 0.8–11)	0.2 (0.7 ± 0.8; 0.1–4.5)
	3 servings: 90 (4.0%)	2.9 (4.1 ± 4.6; 0.1–27)	0.3 (0.7 ± 2.0; 0–13)
	2 servings: 309 (13.8%)	2.6 (3.9 ± 3.8; 1–40.8)	0.2 (0.5 ± 0.9; 0–6.9)
	1 serving: 1567 (69.9%)	2.6 (3.9 ± 3.7; 0.1–35)	0.2 (0.7 ± 1.9; 0–29)
	Never: 227 (10.1%)	2.4 (3.7 ± 3.3; 0.7–25)	0.2 (0.5 ± 0.9; 0–7)
***p*-value**^•^^•^		0.61	0.39
**BMI (kg/m**^2^)	Underweight: 35 (1.6%)	3.0 (4.3 ± 3.4; 1.2–14)	0.5 (1.1 ± 1.4; 0–5.1)
	Normal: 524 (23.4%)	2.7 (3.9 ± 3.4; 0.7–35)	0.2 (0.8 ± 1.8; 0–29)
	Overweight: 715 (31.9%)	2.6 (4.1 ± 4.1; 0.8–32)	0.2 (0.7 ± 1.8; 0–22)
	Obese: 856 (38.2%)	2.5 (3.7 ± 3.4; 0.1–30)	0.2 (0.6 ± 1.5; 0–22)
	Morbid obesity: 113 (5.0%)	2.6 (3.9 ± 4.5; 0.8–40.8)	0.2 (0.7 ± 2.3; 0–22)
***p*-value**^•^^•^		0.04^*^	0.01^*^
**Sun exposure (15 min/day)**	Never: 411 (18.3%)	2.6 (3.9 ± 3.4; 0.8–27)	0.2 (0.6 ± 1.2; 0–13)
	1–2x/week: 492 (21.9%)	2.5 (3.5 ± 2.8; 0.1–25)	0.2 (0.6 ± 1.4; 0–22)
	3–4x/week: 550 (24.5%)	2.7 (4.1 ± 3.9; 0.6–29)	0.2 (0.8 ± 1.8; 0–22)
	≥5x/week: 789 (35.2%)	2.6 (4.1 ± 4.1; 0.1–40.8)	0.2 (0.7 ± 1.9; 0–29)
***p*-value**^•^^•^		0.81	0.76
**Eggs/dairy (yogurt, cheese, labneh)**	Never: 125 (5.6%)	2.6 (3.7 ± 3.4; 0.9–25)	0.2 (0.6 ± 1.4; 0–13)
	1–2x/week: 668 (29.8%)	2.6 (3.8 ± 3.5; 0.1–32)	0.2 (0.6 ± 1.2; 0–15)
	3–4x/week: 790 (35.2%) ≥5x/week: 659 (29.4%)	2.6 (3.9 ± 3.9; 0.6–30)	0.2 (0.7 ± 1.8; 0–22)
		2.7 (3.9 ± 3.7; 0.1–40.8)	0.2 (0.7 ± 1.9; 0–29)
***p*-value**^•^^•^		0.82	0.66
**DEXA scan**	No: 1708 (76.2%)	2.5 (3.6 ± 3.4; 0.1–40.8)	0.2 (0.5 ± 1.2; 0–22)
	Don’t know: 381 (17.0%)	3.0 (4.5 ± 4.4; 0.7–35)	0.3 (1.0 ± 2.6; 0–29)
	Yes: 153 (6.8%)	4.7 (5.7 ± 4.7; 0.6–28)	0.5 (1.4 ± 3.1; 0–22)
***p*-value**^•^^•^		< 0.001*	< 0.001^*^

^•^Mann-WhitneyU ^•^^•^Test The Kruskal-Wallis Test.

*Significant at ≤0.05.

#### The association of protective behaviors with the major osteoporotic fracture risk score among the studied Egyptian participants

The risk of major osteoporotic fracture was significantly higher among participants taking osteoporosis medications other than Calcium/vitamin D (*p* < 0.001), those regularly supplementing with vitamin D or calcium (*p* < 0.001), and those who underwent DEXA scans (*p* < 0.001). These findings represent risk-driven behavior rather than risk-inducing behavior. Higher major fracture risk scores were also observed with increasing BMI (*p* = 0.04) and among participants with different physical activity levels (*p* = 0.004). In contrast, daily milk intake, egg/dairy consumption, and sun exposure showed no significant association with major fracture risk (Table 4).

#### The association of protective behaviors with hip osteoporotic fracture risk score among the studied Egyptian participants

Hip fracture risk showed similar associations, being significantly elevated in participants taking osteoporosis medications (*p* < 0.001), vitamin D and calcium supplements (*p* < 0.001), and those undergoing a DEXA scan (*p* < 0.001). These findings represent risk-driven behavior rather than risk-inducing behavior. Also, Hip fracture risk was significantly elevated with higher BMI (*p* = 0.01). Physical activity was inversely related to hip FRAX (*p* < 0.001). Conversely, milk consumption, egg/dairy intake, and sun exposure were not significantly associated with hip fracture risk (Table 4).

### The classification of FRAX scores and their correlation with the OKAT score among Egyptian participants

**Fig. 1:**
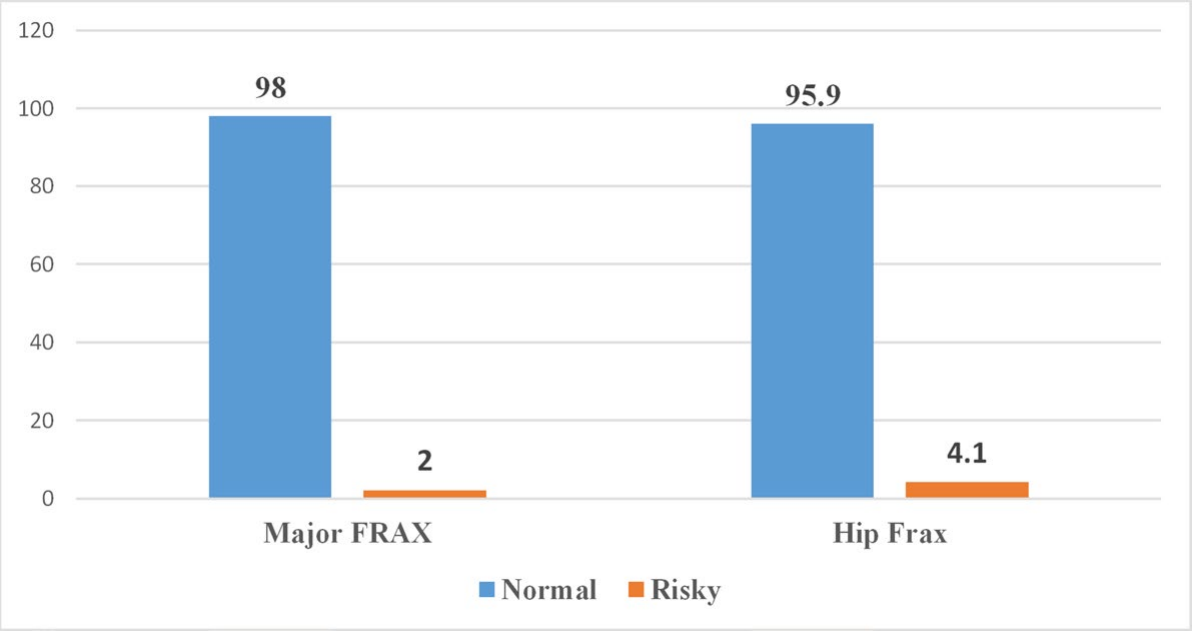
**The distribution of Major and Hip fracture risk among the studied adult Egyptians.**

Major fracture: Most participants (98%) were categorized as Normal, while only 2% were considered at high risk. Hip fracture: Similarly, 95.9% of participants were classified as Normal, with 4.1% falling into the high-risk category (Fig. 1).

The correlation analysis revealed that the OKAT score was negatively correlated with both major osteoporotic fracture and hip fracture risks, but the association reached statistical significance only with hip fracture (*p* = 0.007). A strong and highly significant positive correlation was found between major osteoporotic and hip fractures (*r* = 0.69, *p* < 0.001), indicating that participants at higher risk of major fractures were also more likely to be at risk of hip fractures (Table 5).

**Table 5:** **The correlation between the OKAT, major, and hip osteoporotic fractures scores among the Egyptian participants.**

The variables		r(*P*)
OKAT score	Major Osteoporotic Fracture	−0.33(0.14)
OKAT score	Hip Fracture	−0.06(0.007^*^)
Major Osteoporotic Fracture	Hip Fracture	0.69(< 0.001^*^)

^*^Spearman’s correlation is significant at *p *≤ 0.01.

**Fig. 2:**
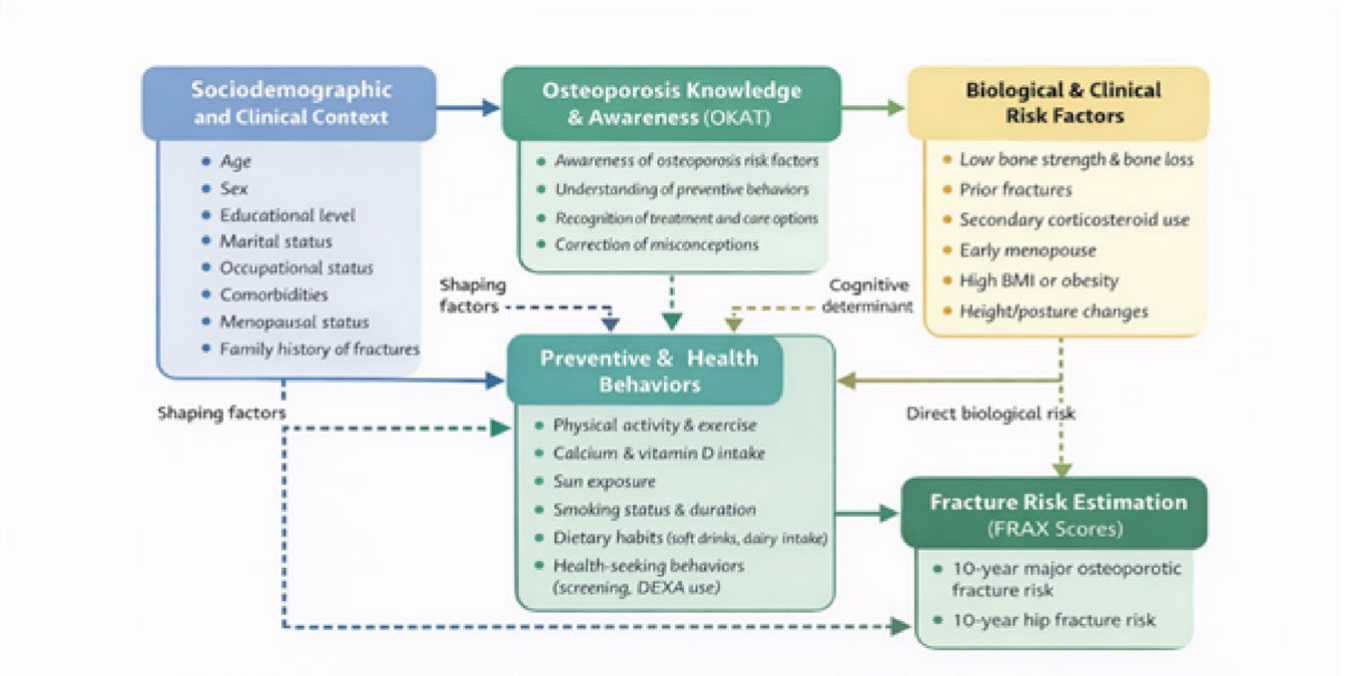
**Conceptual framework linking sociodemographic factors, osteoporosis knowledge, preventive behaviors, and fracture risk.**

**Fig. 3:**
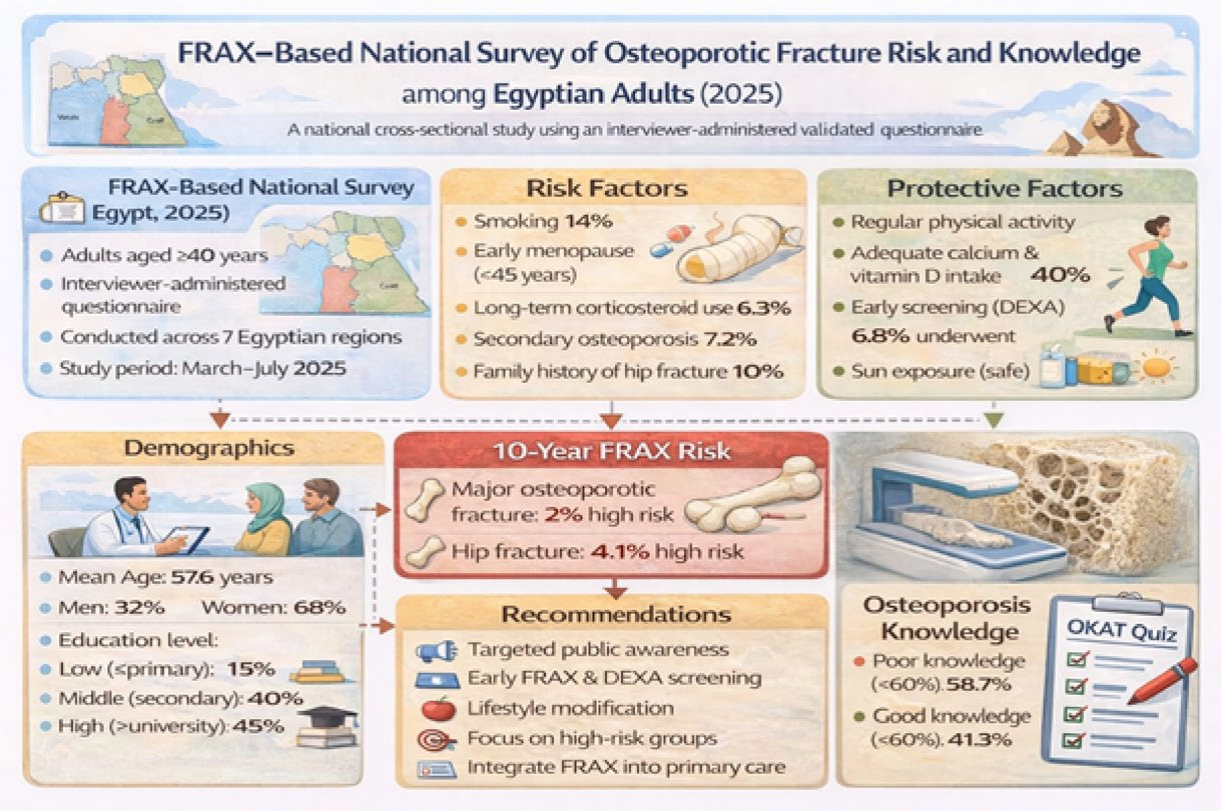
**Understanding Osteoporotic Fracture Risk in Egypt: Key Findings from a National FRAX Survey (2025)**. Created in BioRender. Moghib, K. (2026) https://BioRender.com/0c5vxp0.

Based on the observed associations between sociodemographic characteristics, osteoporosis knowledge, preventive behaviors, and FRAX scores, a conceptual framework was developed to illustrate the hypothesized pathways linking these domains (Fig. 2). Moreover, a comprehensive infographic for Understanding Osteoporotic Fracture Risk in Egypt: Key Findings from a National FRAX Survey (2025) (Fig. 3).

## Discussion

Osteoporosis remains a pressing global public health challenge, and its impact in Egypt is poised to grow in tandem with an aging population and shifting lifestyle patterns. Understanding the factors associated with osteoporotic fracture risk, as well as the community’s level of knowledge, is essential for shaping effective prevention and early intervention strategies^1^.

### The associations between demographic determinants and the OKAT score

**In this study**,** over half of the participants were found to have limited knowledge about osteoporosis**,** e**choing earlier findings from Egypt and the region, where poor knowledge rates ranged from 46.6% to 58.5%^2,13^. Notably, women demonstrated significantly greater awareness than men, consistent with prior research^14^. This difference likely reflects the higher burden of osteoporosis in women, particularly postmenopausal women, and their increased exposure to health education and screening initiatives.

**Educational attainment emerged as another key factor: participants with higher education levels showed better knowledge about osteoporosis**,** supporting previous findings**^2^. This may be due to their greater access to and ability to interpret health information through formal education, media, and digital resources. Interestingly, in contrast to some earlier studies^2^, age in this study was also significantly linked to knowledge levels, which may stem from variations in sample demographics, educational background, or public health outreach.

**While most participants recognized primary risk factors and preventive strategies—**such as the importance of physical activity and fracture risk—misconceptions persisted. These included misunderstandings about the role of salt intake, ethnicity, and treatment options, as documented in prior research^2^. Such knowledge gaps likely arise because public health messaging frequently emphasizes factors like aging, menopause, and calcium, while placing less focus on dietary salt, treatment effectiveness, or epidemiological nuances.

***Regarding knowledge of hormonal therapy and other misconceptions***, 59.4% and 53.8% of our participants did not recognize the preventive role of hormonal therapy in postmenopausal bone loss and bone loss within 10 years, respectively. Misconceptions about osteoporosis were common: 74.4% incorrectly believed it causes symptoms before fractures, 46.3% thought it is more common in men, and 56.7% correctly identified that calcium alone cannot prevent bone loss. In^2^, we observed similar patterns. These patterns may reflect cultural beliefs, population characteristics, and lifestyle factors that influence awareness and acceptance of hormonal therapy as a preventive measure. Unfortunately, misconceptions may lead to an underestimation of personal risk and delayed medical evaluation. Osteoporosis is often referred to as a “silent disease” because it is asymptomatic until fractures occur; therefore, believing that symptoms such as back pain or joint stiffness are always present can result in false reassurance and late detection.

### The Association between demographic determinants and the osteoporotic fracture risk score

***This study found a significant association between marital status and fracture risk;*** widowed and divorced individuals exhibited higher risks for major osteoporotic and hip fractures compared to those who were married, supporting earlier evidence^15^. Reduced social support, increased psychological stress, and the challenges of living alone may result in lower engagement with preventive behaviors, higher fall risk, and delayed healthcare.

***Age showed a strong and graded association with fracture risk***, a pattern confirmed by Egyptian national registry data indicating a pronounced increase in hip fracture rates after age 50^16^, as well as established mechanisms underlying age-related osteoporosis^17^. Additionally, lower education and occupational statuses, especially manual labor and retirement, were linked to increased fracture risk, echoing findings from other populations and illustrating the importance of socioeconomic factors^18^.

***Several comorbidities***, including rheumatoid arthritis, renal disease, and thyroid disorders, were found to raise fracture risk. Unlike some studies that identified no link between the overall burden of comorbidities and fracture occurrence^19,20^, our results suggest that certain chronic conditions with direct effects on bone metabolism may play a more pivotal role than the total number of comorbidities.

### Osteoporosis Risk Factors and their association with the osteoporotic fracture risk score among the studied Egyptian participants

The most common risk factor was a history of previous fractures, affecting more than a third of the participants; this may be attributed to the highly prevalent lifestyle factors, such as smoking and frequent consumption of soft or energy drinks, that support these fractures. As well as women, early menopause and contraceptive use were reported, both of which may impact bone health depending on hormonal exposure.

Moreover, secondary osteoporosis, long-term corticosteroid use, and a family history of hip fracture. These findings align with previous studies that identified smoking, diabetes, and prior fractures as key risk contributors^20^, as well as research highlighting the role of endocrine and metabolic factors^21^.

***Notably***,*** prolonged corticosteroid use was associated with higher major and hip fracture risk*** (*p* < 0.001), which contrasts with some cohort studies that did not observe such associations^19,22^. This difference could be influenced by variations in clinical practice, patient characteristics, or the use of concurrent bone-protective therapies in certain settings.

***Smoking was also linked to higher fracture risk;*** particularly with longer exposure, supporting the established causal relationship between tobacco use and osteoporotic fractures^23,24^. Higher fracture risk was also noted among individuals with secondary osteoporosis, prior fractures, early menopause, and a family history of hip fracture, further underlining the multifactorial nature of osteoporosis risk^25–27^.

***Hormone replacement therapy was not significantly associated with major FRAX*** ***scores;*** in the present study; however, longer duration of therapy correlated with lower fracture risk, supporting evidence that benefits are greatest when therapy is started near menopause and continued over time^25^. The absence of data regarding timing and continuity of therapy in this study may have limited the observed associations^28^.

***Alcohol intake***; meanwhile, was not significantly associated with fracture risk, mirroring earlier findings^29^. This likely reflects the very low prevalence of alcohol use in this population, which in turn limits variability and the ability to detect associations.

### Protective behaviors and their association with the osteoporotic fracture risk score among the studied Egyptian participants

***Despite the well-established importance of vitamin D and calcium for bone health***,*** supplementation rates were relatively low among participants;*** Interestingly, those who did take supplements had higher fracture risk, likely reflecting indication bias: individuals with recognized or perceived risk are more likely to be advised to start supplementation^30–32^. Intake of dairy products and sun exposure did not show significant associations with fracture risk, consistent with previous reports^30,33–35^, and potentially influenced by reporting bias and variability in vitamin D synthesis.

***Eggs***,*** Milk***,*** Dairy Diet and its association with the major osteoporotic fracture risk among the Egyptian population***; The Egyptian participants had inadequate bone-supportive diets. According to regional research, Arab adults consume less calcium-rich food, as nearly 70% drink one serving of dairy every day and 10.1% do not. Due to dietary reporting errors and discrepancies, previous research revealed no direct link between dairy intake and fracture risk, despite calcium’s importance in bone health^30,34^. The Egyptian participants’ poor calcium consumption and supplementation raise bone-health vulnerability.

Daily milk intake, egg/dairy consumption, and sun exposure showed no significant association with major fracture risk, which agreed with^35^, where there was no association between Ca intake from dairy products and risk of osteoporosis. Biases and errors in dietary data collection are partly to blame for these discrepancies in the studies on a possible association between milk intake and bone health.

**Physical activity;** on the other hand, was inversely associated with fracture risk, reinforcing its established role in maintaining bone strength, muscle mass, and balance, as well as in fall prevention^36,37^.

***Sunlight and Exercise and its association with the major osteoporotic fracture risk among the Egyptian population;*** Middle Easterners were sedentary, with 41.9% not exercising^36^. Inactivity reduces bone strength, muscle mass, and balance, preventing fractures. In the Saudi Arabian safe solar exposure trial, awareness did not influence behavior, and 18.3% avoided daily sunlight. Vitamin D production declines with insufficient sunlight; however, clothing, pigmentation, season, and cutaneous UVB absorption may obscure its relationship to fracture reduction. Vitamin D deficiency and sun avoidance can lead to osteoporosis.

In contrast, sun exposure alone was not associated with fracture risk, likely due to unmeasured factors such as cultural clothing, skin pigmentation, timing of exposure, and sunscreen use, all of which can substantially affect vitamin D status and are particularly relevant in Middle Eastern populations^38,39^.

***Higher body mass index (BMI) was associated with increased fracture risk***. This finding suggests that excess weight may contribute to risk via impaired balance, reduced mobility, and higher impact forces during falls, even though some meta-analyses have reported an inverse relationship between BMI and fracture risk^40^. The complex interplay between BMI and bone health may depend on a variety of factors, including distribution of body fat and differences in physical activity.

***Among those who underwent DEXA scanning***, fracture risk was higher, likely reflecting indication bias: individuals perceived to be at greater risk are more likely to undergo diagnostic evaluation. This supports the use of FRAX as a triage tool to optimize patient selection for DEXA scanning^41^.

### The classification of FRAX scores and their correlation with the OKAT score among Egyptian participants

Only a small proportion of study participants were identified as high risk for major osteoporotic or hip fractures. This may be due to the relatively younger age profile of the sample and the known limitations of FRAX, which can underestimate risk in younger or obese individuals when bone mineral density data are not included^42,43^. By contrast, studies among postmenopausal women with osteopenia have reported much higher risk, reflecting differences in study populations and design.

A modest inverse correlation was observed between osteoporosis knowledge (OKAT scores) and FRAX-based fracture risk, with a statistically significant association for hip fracture risk. To our knowledge, this is the first study to explore the relationship between osteoporosis knowledge and objectively assessed fracture risk, highlighting the potential impact of education and awareness on osteoporosis prevention.

### Strengths and limitations

This research represents the first comprehensive, nationwide survey in Egypt to assess osteoporosis knowledge, risk factors, preventive behaviors, and fracture risk using validated instruments such as FRAX and OKAT. The large and diverse sample enhances the generalizability and public health relevance of the findings.

**Nevertheless**,** several limitations** should be acknowledged. The cross-sectional design precludes causal inference, and the use of convenience sampling may limit the representativeness of the sample as it may introduce selection and clustering bias. Reliance on self-reported data introduces the potential for recall and social desirability bias. The statistical analysis was limited to descriptive and bivariate methods, as multivariable modeling was not feasible given the low prevalence of high-risk FRAX categories, which could undermine model stability.

Although the OKAT has been validated in Arabic-speaking populations, cultural perceptions may influence responses to certain items, particularly those related to treatment availability. However, retaining all validated items allows identification of knowledge gaps and ensures comparability across studies.

## Conclusion

This national survey revealed that only a trivial proportion of the studied adult Egyptians over 40 years old were at high risk for major and hip fractures. However, more than half of the participants had poor knowledge, significant knowledge gaps, and misconceptions regarding osteoporosis. A high prevalence of osteoporotic fracture risk factors and inadequate preventive practices was evident.

Moreover, the OKAT score was significantly negatively correlated with hip fracture risks and insignificantly with major osteoporotic fractures. A strong and highly significant positive correlation was found between major osteoporotic fractures and hip fractures.

## Recommendations

- ***Develop nationwide Public Educational Campaigns or Interventions*** utilizing the expertise of both healthcare professionals and reliable media sources that specifically target high-risk groups (smokers, those with early menopause, chronic corticosteroid users, and individuals with a family history of fractures) to address misconceptions and knowledge gaps, the importance of early detection (DEXA screening), and preventive interventions to reduce the burden of osteoporosis through community programs.
- ***Promoting healthy lifestyles should*** include regular physical activity, a balanced diet, safe sun exposure, and adequate intake of vitamin D and calcium.

- ***Future research***,*** particularly longitudinal studies***, should integrate data on bone mineral density characterized by a more equitable age distribution, elevated event rates, and balanced risk distributions. This would provide strong multivariable modeling and the validation of independent predictors, thus enhancing the evidence base for osteoporosis prevention, investigating causal linkages, and assessing the efficacy of intervention programs.
- ***Integrating brief osteoporosis education training courses targeted at health care professionals***, along with FRAX screening, into routine primary care visits for adults over 40 could facilitate the early identification of high-risk individuals.
- ***Expand Access to osteoporosis screening***: Increase affordability and availability of DEXA scans in all of Egypt, both in urban and rural areas.

## Supplementary Information

Below is the link to the electronic supplementary material.

Supplementary Material 1

## Data Availability

All data supporting the research findings are available from the corresponding author upon request from the editor.

## **Abbreviations**

BMD: Bone Mineral Density
DXA: Dual X-ray Absorptiometry
FRAX: Fracture Risk Assessment
HF: Hip Fracture
MOF: Major Osteoporotic Fracture
OKAT: Osteoporosis Knowledge Assessment Test
UVB: Ultraviolet B
