# Supplementary material for: FRAX-based national survey of osteoporotic fracture risk, related knowledge, and associated factors, among Egyptian adults aged 40 years and older, 2025
Source: Sci Rep. 2026 Apr 29;16:13767. doi: 10.1038/s41598-026-40443-4 (PMC13129074; doi:10.1038/s41598-026-40443-4)
Supplement: Supplementary file 1 — Supplementary Material 1 [file 41598_2026_40443_MOESM1_ESM.pdf]

Fwd: طلب استبيان

From: Doaa Omar (doaaibrahim8282@yahoo.com)

To: dr_samar11@yahoo.com

Date: Tuesday, January 27, 2026 at 08:38 PM GMT+3

Sent from my iPhone

Begin forwarded message:

> **From:** Doaa Omar <doaaibrahim8282@yahoo.com>
> **Date:** 29 March 2025 at 2:17:05 AM GMT+2
> **To:** "Dr. Maha Wahdan" <drmaha_wahdan@med.asu.edu.eg>
> **Subject: Re:** طلب استبيان

شكرا جزيلا لحضرتك د مها
جزاك الله خيرا
هل الarabic version دي هي اللي تم عمل validation لها في دراسة سابقة sayed Hassan et al
ام حضراتكم اللي ترجمتوها ؟؟؟

شكرا جزيلا ❤️❤️

On 28 Mar 2025, at 5:25 PM, Dr. Maha Wahdan <drmaha_wahdan@med.asu.edu.eg> wrote:

https://docs.google.com/forms/d/1h2q6ztKrJq0SbX1qDzEcG0r39kNqt5V9n7CO6HG3lbw/edit

السلام عليكم
اسفه علي التاخير في الرد
ده لينك الاستبيان لاني مش لاقيه الملف الوورد النهائي

ربنا يوفقك

From: Doaa Omar <doaaibrahim8282@yahoo.com>
Sent: Tuesday, March 11, 2025 12:14 AM
To: Dr. Maha Wahdan <drmaha_wahdan@med.asu.edu.eg>
Subject: طلب استبيان

السلام عليكم د/ مها وهدان مع حضرتك د/دعاء ابراهيم أستاذ مساعد الصحة العامة نظرا للتحضير لعمل دراسة عن الosteoporosis risk and perceptions وجدت البحث الخاص بكم بعنوان

Knowledge about Symptoms and Risk Factors of Osteoporosis among Adult Women in Cairo, Egypt

وقد تم استخدام OKAT questionnaire

فهل يمكن ارسال النسخة العربية من الاستبيان للضرورة لاستخدامها مع التأكيد علي ذكر البحث الخاص بكم كمصدر للاستبيان 🌹🌹

وشكرا مقدما 🌹🌹

CAUTION: This email originated from outside of the organization. Do not click links or open attachments unless you recognize the sender and know the content is safe.
